# Mono‐dimensional, two‐dimensional and Doppler echocardiographic measurements in healthy Standardbred neonatal foals in the first 5 days of life

**DOI:** 10.1002/evj.70140

**Published:** 2026-01-06

**Authors:** Fernanda Timbó D'el Rey Dantas, Giulia Forni, Gayle Hallowell, Carolina Castagnetti, Laura Menchetti, Giovanni Romito, Aliai Lanci, Jole Mariella, Francesca Freccero

**Affiliations:** ^1^ Department of Veterinary Medical Sciences University of Bologna Bologna Italy; ^2^ Department of Animal Medicine, Production and Health University of Padova Padova Italy; ^3^ Medicine Vet Equine Referrals Leicestershire UK; ^4^ School of Biosciences and Veterinary Medicine University of Camerino Camerino Italy

**Keywords:** cardiovascular adaptation, haemodynamic assessment, horse, left ventricular function, newborn, postnatal development

## Abstract

**Background:**

Bodyweight, age and breed influence the echocardiographic assessment of foals. There are no echocardiographic studies in Standardbred neonatal foals.

**Objectives:**

To describe echocardiographic values for selected variables, evaluate intra‐ and inter‐observer variability and assess cardiac changes in the first 5 days of life in healthy Standardbred neonatal foals.

**Study Design:**

Prospective observational study.

**Methods:**

Fifty‐six healthy Standardbred neonatal foals were examined by transthoracic echocardiography using standard right parasternal and subcostal views at three time points: in the first 48 h (T1), between 49 and 96 h (T2), and 97 and 144 h (T3) after birth. Descriptive statistics, variability analysis and linear mixed models assessed age‐related changes in cardiac parameters. Intra/inter‐observer variability was assessed using intraclass correlation coefficients (ICC).

**Results:**

A total of 114 echocardiographic examinations were performed. Intra‐observer agreement was excellent for most variables, while inter‐observer agreement was excellent in approximately half. An increase in end‐diastolic left ventricular internal diameter (T1: 5.3 ± 0.6 cm; T3: 5.6 ± 0.8 cm; *p* < 0.01), left atrial diameter in the four‐chamber view (T1: 5.8 ± 0.6 cm; T3: 6.0 ± 0.6 cm; *p* = 0.01), end‐diastolic aortic sinus diameter in the left ventricular outflow tract view (T1: 3.0 ± 0.3 cm; T3: 3.2 ± 0.3 cm; *p* < 0.01) and peak velocity of the transmitral E wave (T1: 0.8 ± 0.1 m/s; T3: 0.9 ± 0.1 m/s; *p* < 0.01) were observed over time. Additionally, a gradual decrease in the end‐diastolic pulmonary diameter at the sinus (T1: 2.8 ± 0.3 cm; T3: 2.5 ± 0.3 cm; *p* < 0.01) and at the valve (T1: 2.6 ± 0.3 cm; T3: 2.5 ± 0.4 cm; *p* = 0.03) levels was noted.

**Main Limitations:**

Some variables have fewer individuals; coefficients of variation are moderate to high for some variables.

**Conclusions:**

Changes in echocardiographic variables in a healthy Standardbred neonatal foal population reflect a physiological adaptation of the cardiorespiratory system from foetal to extrauterine life. These observations can be used as a reference in the assessment of neonatal foals of similar age and weight.

## 1 INTRODUCTION

Echocardiography is a widely used diagnostic technique in equine medicine and serves as the primary method for cardiac imaging. In equine neonatology, it has been used not only to unveil the presence of congenital cardiac defects,^1^, ^2^ but also to document the development of cardiovascular complications (such as pulmonary hypertension, endocarditis and myocarditis) as well as assess the cardiac function and fluid responsiveness in critically ill foals.^3^, ^4^, ^5^ To correctly interpret echocardiographic findings in foals, reference values from healthy subjects comparable in age, bodyweight and breed are necessary, as these and other physiological features (such as the relatively larger right ventricle in the first hours) remarkably impact echocardiographic variables.^2^, ^6^, ^7^ In addition, foals have anatomical particularities that influence in echocardiographic assessment, such as the smaller thorax that allows for the use of the subcostal view.^8^

Reference ranges for several echocardiographic measurements have been reported for different breeds of adult horse.^9^, ^10^, ^11^ However, these parameters have been investigated only in pony,^12^, ^13^ Thoroughbred,^7^, ^13^, ^14^ Arabian^15^ and Spanish^16^ foals. Therefore, studies regarding echocardiographic measurements of healthy Standardbred neonatal foals are currently lacking.

The aims of the present study were: (1) to describe measurement values for selected M‐mode, two‐dimensional (2DE) and Doppler variables in healthy Standardbred neonates; (2) to evaluate the intra‐ and interobserver variability of these measurements; (3) to assess the changes in these echocardiographic parameters in the first 5 days of life in healthy foals. We hypothesised that echocardiographic parameters of cardiac size and function would present changes during the first days of life, reflecting the physiological transition from foetal to postnatal circulation and foals' growth.

## 2 MATERIALS AND METHODS

### 2.1 Study design

Prospective, observational, single‐centre study.

### 2.2 Animals and inclusion criteria

Fifty‐six Standardbred foals (*n* = 26 males, *n* = 30 females) born from mares that were admitted for attended parturition to the Equine Perinatology and Reproduction Unit of the Department of Veterinary Medical Sciences of the University of Bologna during four foaling seasons (2020, 2021, 2022 and 2023) were included. All foals were of the same breed (Standardbred); detailed information regarding genetic diversity was not available, although it is known that multiple stallions were used on each farm. Foals were considered healthy if: they were born at term (more than 320 days of pregnancy) after a normal pregnancy and parturition; presented an APGAR score ≥8 at birth^17^; had normal haematology and biochemistry at birth as well as a serum IgG at 12–24 h of life >8 g/L (immunoturbidimetric method; DVM Rapid Test II, MAI Animal Health); and had an unremarkable physical examination, including a normal heart rate and rhythm (72–150 bpm)^18^ and no pathological murmurs on cardiac auscultation throughout the study period.^17^ All foals were housed with their dams, allowed to nurse freely, with daytime access to a paddock. They were examined daily during the observation period at the hospital and were discharged 5–7 days after birth.

### 2.3 Study protocol and echocardiographic examination

All foals underwent a complete transthoracic echocardiographic examination, including M‐mode, 2DE and Doppler evaluation, at three time points: in the first 48 h (T1), between 49 and 96 h (T2), and 97 and 144 h (T3) after birth. Exact age (in hours) and bodyweight (kg) were recorded at each time point.

Echocardiographic examination was performed using a standard right parasternal^2^, ^19^ and a subcostal view^8^ to avoid changing recumbency. Foals were positioned in left lateral recumbency on a mattress placed in front of the stall so that the broodmare was always in view. They were restrained by trained personnel; no pharmacological sedation was employed. The right forelimb was extended slightly cranially to improve echocardiographic images. No clipping was employed; alcohol and ultrasound gel were used for image acquisition as needed.

All echocardiographic exams were performed by the same operator (FF). Two different ultrasound systems were used: a MyLab Alpha device (Esaote Lab) during the first two foaling seasons (2020 and 2021) and a Philips CX50 system (Philips Medical Systems, USA) during the last two (2022 and 2023), both equipped with 1–5 MHz phased array cardiologic transducers. Images were acquired using second harmonic imaging. Each foal was consistently examined with the same ultrasound system throughout the study, and no animal was assessed with different devices. A single lead base‐apex electrocardiogram was recorded during each echocardiographic examination.

Routine transthoracic M‐mode, 2DE and Doppler echocardiography were performed to assess cardiac structures, chamber dimensions, LV systolic function and transvalvular flows as described elsewhere.^2^, ^19^ All echocardiographic recordings were stored in DICOM format as still frames or cine‐loops for offline analysis performed at the end of the data collection period. Echocardiographic measurements were performed offline using the built‐in software of the ultrasound machine by blinded observers. For each variable, the mean of three measurements from three different cardiac cycles was used for further analysis.

### 2.4 Echocardiographic measurements and indices

Details about echocardiographic measurements and indices, including scan views, methods, abbreviations and units can be found in Table S1.

### 2.5 Inter‐ and intra‐observer reliability

To assess measurement reliability, six foals were randomly selected from the study cohort, following a statistical plan based on a 5% error probability, a 90% confidence interval with a width of 0.15 and an estimated diagnostic error rate of 0.05. Randomisation was performed using the RAND.BETWEEN function in Microsoft Excel. For each animal, a single echocardiographic examination was chosen, resulting in a total of six different studies being analysed. For interobserver variability, two observers with different levels of echocardiographic experience, namely a Diplomate of the European College of Equine Internal Medicine (FF) and a PhD student (FTDRD), independently measured all variables. To assess inter‐day intra‐observer variability, the same observer (FTDRD) repeated the measurements for each of the 6 foals on different days, with at least a 7‐day interval. Care was taken to analyse the same images. Intra‐observer and inter‐observer reliability were expressed as Intraclass Correlation Coefficients (ICC) using the two‐way mixed‐effect model for single measurement and absolute agreement. ICC values were interpreted as poor (ICC < 0.40), fair (0.40 ≤ ICC < 0.60), good (0.60 ≤ ICC < 0.75) and excellent (ICC ≥ 0.75).^20^

### 2.6 Data analysis

The sample size was calculated to detect a statistically significant difference across three repeated measures within a single group. Using G*Power software (version 3.1.9.7), a repeated‐measures ANOVA was planned based on an expected medium effect size (Cohen's *f* = 0.25), a significance level (*α*) of 0.05 and a statistical power (1 − *β*) of 0.95. The calculation also considered a moderate correlation between repeated measures (*r* ≈ 0.5). This analysis indicated that a minimum of 43 animals would be required to adequately power the study and minimize the risk of a Type II error. In practice, 56 horses met the predefined inclusion criteria across the four foaling seasons, and all were included to maximize statistical power and reduce the risk of Type II error, while improving the precision of the estimates. The post‐hoc analysis indicated that a statistical power of 98.7% was achieved.

Extreme outliers, defined as values more than three interquartile ranges above the upper quartile or below the lower quartile, were identified across the entire dataset using diagnostic plots and subsequently excluded. In particular, one outlier was eliminated for heart rate, R‐4C LVLs and LVFWd variables. Descriptive statistics were shown for three age ranges: 0–47 h, 48–97 h and 98–148 h. The Kolmogorov–Smirnov test assessed the normality of each variable's data distribution. Although most parameters followed a normal distribution, a robust approach based on the 5th and 95th sample quantiles was adopted to determine the reference limits. Additionally, bootstrap resampling was used to calculate the 90% confidence intervals of these limits. Moreover, mean ± standard deviation (SD), median [interquartile range (IQR)] and inter‐individual coefficient of variation (CoV) were reported.^21^ To interpret the CoV, the degree of variability was categorised as follows: CoV <5% indicated very low variability; 5%–15% low variability; 16%–25% moderate variability and >25% high variability.^22^

Prior to performing inferential statistical analyses, some variables (i.e., R‐LVVdSAX, R‐SV_Bullet_SAX, LVIDd, EDV_M‐mode_SAX and SV_M‐mode_SAX) were natural log‐transformed (ln) to improve their distribution. The data were subsequently analysed using linear mixed models with a first‐order autoregressive [AR (1)] covariance structure. Foal ID was included as a random effect and subject identifier, while age (in hours) was treated as a within‐subjects variable.^23^ The models assessed the effect of age, entered as a continuous predictor. For each model, the regression coefficient (b), representing the change in the dependent variable per 1‐h increase in age, and the corresponding *p*‐value were reported.

Statistical analyses were performed with SPSS Statistics version 25 (IBM, SPSS Inc.). Statistical significance occurred when *p* < 0.05.

## 3 RESULTS

A total of 114 echocardiographic examinations in 56 foals were performed in this study. Seventeen foals (30%) were examined at three time points, 24 (43%) at two time points and 15 (27%) at only one time point. The frame rate during Doppler recordings ranged from 12 to 28 frames/s. Neither congenital nor acquired cardiologic abnormalities were observed in this foal population. The mean ± standard deviation age (hours) for T1, T2 and T3 was 19 ± 7, 75 ± 14 and 117 ± 13, respectively. The mean ± standard deviation bodyweight (kg) was 50.0 ± 5.4, 54.1 ± 6.0 and 56.5 ± 5.6 for T1, T2 and T3, respectively. The heart rate ranged from 60 to 138 bpm. The mean ± standard deviation heart rate (bpm) in T1, T2 and T3 was 88 ± 11, 89 ± 13 and 89 ± 16, respectively. The duration of examinations varied between years, with a mean of 23 min per foal.

Intra‐observer agreement was excellent for most variables (29/37), although R‐PAD_valve_SAX and R‐PAD_sinus_SAX exhibited poor agreement. Similarly, inter‐observer agreement was excellent in just over half of the variables (22/37), while R‐LAD1dSAX, R‐LAAdSAX, R‐PAD_valve_SAX, R‐PAD_sinus_SAX and R‐4C TV_A_ presented poor agreement. Detailed results of both intra‐ and interobserver agreement are presented in Table 1. Variables that showed intra‐ or inter‐observers' ICC <0.60 did not have reference limits reported.

**TABLE 1:** Intra‐ and interobserver reliability of echocardiographic parameters in healthy neonatal foals based on intraclass correlation coefficients (ICC) with lower and upper 95% confidence intervals (CI) and respective *p*‐values.

Parameter	Intra‐observer				Inter‐observer			
	ICC	95% CI	95% CI	*p* value	ICC	95% CI	95% CI	*p* value
		Lower bound	Upper bound			Lower bound	Upper bound	
R‐4C LVVd	0.81	0.15	0.97	0.02	0.62	−0.10	0.93	0.03
R‐4C LVVs	0.80	0.14	0.97	0.02	0.54	−0.14	0.91	0.06
R‐4C LVLd	0.92	0.52	0.99	<0.01	0.72	0.00	0.96	0.04
R‐4C LVLs	0.96	0.33	1.00	<0.01	0.88	0.22	0.98	<0.01
R‐LVAdSAX	0.99	0.95	1.00	<0.01	0.98	0.86	1.00	<0.01
R‐LVAsSAX	0.95	0.68	0.99	<0.01	0.94	0.66	0.99	<0.01
R‐LVVdSAX	0.99	0.94	1.00	<0.01	0.95	0.66	0.99	<0.01
R‐LVVsSAX	0.95	0.67	0.99	<0.01	0.93	0.63	0.99	<0.01
RVIDd	0.57	−0.50	0.93	0.12	0.84	0.33	0.98	0.01
IVSd	0.87	0.28	0.98	0.01	0.86	0.39	0.98	0.01
IVSs	0.98	0.88	1.00	<0.01	0.92	0.39	0.99	<0.01
LVIDd	0.92	0.60	0.99	<0.01	0.96	0.20	1.00	<0.01
LVIDs	0.99	0.87	1.00	<0.01	0.84	−0.03	0.98	<0.01
LVFWd	0.97	0.83	1.00	<0.01	0.99	0.95	1.00	<0.01
LVFWs	0.98	0.88	1.00	<0.01	0.90	0.48	0.99	<0.01
EPSS	0.94	0.03	0.99	<0.01	0.55	−0.14	0.92	0.05
R‐4C LAD	0.56	−0.15	0.92	0.07	0.86	0.25	0.98	0.01
R‐4C LAA	0.80	0.21	0.97	0.01	0.86	0.40	0.98	0.01
R‐LAD1dSAX	0.70	0.01	0.95	0.03	0.30	−0.13	0.81	0.08
R‐LAD2dSAX	0.84	0.18	0.98	0.01	0.59	−0.17	0.93	0.07
R‐LAAdSAX	0.73	0.02	0.96	0.01	0.27	−0.06	0.79	0.03
R‐AoD_valve_LVOT	0.91	0.53	0.99	<0.01	0.75	0.10	0.96	0.02
R‐AoD_sinus_LVOT	0.95	0.70	0.99	<0.01	0.95	0.39	0.99	<0.01
R‐AoD_STJ_LVOT	0.96	0.73	0.99	<0.01	0.82	0.25	0.97	0.01
R‐PADdLVOT	0.92	0.23	0.99	<0.01	0.80	−0.01	0.97	<0.01
R‐PAD_valve_RVOT	0.97	0.86	1.00	<0.01	0.56	−0.12	0.92	0.01
R‐PAD_sinus_RVOT	0.98	0.85	1.00	<0.01	0.92	0.27	0.99	<0.01
R‐AoDdSAX	0.99	0.93	1.00	<0.01	0.69	−0.05	0.96	<0.01
R‐PAD_valve_SAX	0.10	−1.00	0.82	0.43	0.09	−0.11	0.60	0.29
R‐PAD_sinus_SAX	0.39	−0.60	0.89	0.21	0.24	−0.11	0.77	0.09
SC AoD_sinus_	0.87	0.39	0.98	<0.01	0.76	−0.01	0.96	0.03
R‐PA Vel_max_ RVOT	0.99	0.96	1.00	<0.01	0.95	0.63	0.99	<0.01
R‐PA Vel_max_ SAX	0.41	−0.66	0.90	0.20	0.69	−0.23	0.95	0.06
R‐4C TV_E_	0.82	0.11	0.97	0.02	0.67	−0.03	0.94	0.04
R‐4C TV_A_	0.42	−0.30	0.88	0.15	0.36	−0.22	0.85	0.14
SC Ao Vel_max_	0.94	0.50	0.99	<0.01	0.64	−0.06	0.95	<0.01
SC Ao VTI	0.86	−0.04	0.98	<0.01	0.86	−0.32	0.98	<0.01

*Note*: Abbreviations and imaging and measurement details are available in Table S1.

Not all variables were measured in every foal; some measurements were unavailable due to poor quality of 81 images (~4% of the total number of images). Further details are provided in Table 2, together with descriptive statistics and linear mixed models analysis of M‐Mode, 2DE and Doppler measurements. Overall, most left ventricular dimensions, left atrial size, aortic diameters, pulmonary and aortic flow and stroke volume measurements increased over time. In contrast, pulmonary artery diameters measured at different levels progressively decreased. Most cases showed low or very low CoV. Moderate CoV was also observed in several cases, while high CoV was present in a slightly smaller proportion.

**TABLE 2:** Descriptive statistics and age‐related changes in echocardiographic parameters across three postnatal time points (T1: 0–47 h, T2: 48–97 h, T3: 98–148 h) in healthy Standardbred foals, including measurement units, number of animals accounted for each parameter (*n*), reference limits, inter‐individual coefficient of variation (CoV), and linear mixed model results.

Parameter	Unit	*n* [T1, T2, T3]	T1 mean ± SD	T1 median [IQR]	T1 reference limits (90%)	T1 CoV (%)	T2 mean ± SD	T2 median [IQR]	T2 reference limits (90%)	T2 CoV (%)	T3 mean ± SD	T3 median [IQR]	T3 reference limits (90%)	T3 CoV (%)	Linear mixed models		
															*b* estimate	SE of *b*	*p*‐value
**Ventricle**																	
**R‐4C LVVd**	**ml**	**46, 37, 31**		**154.40 [133.0–186.7]**	**152.30–170.2**	**23**		**187.7 [150.2–211.8]**	**174.0–199.1**	**25**		**197.7 [162.3–233.0]**	**185.7–213.4**	**23**	**0.314**	**0.048**	**<0.01**
R‐4C LVVs	ml	46, 37, 31		60.3 [51.5–85.2]	^a^	36		64.6 [43.8–89.2]	^a^	42		69.7 [58.0–96.9]	^a^	34	0.046	0.028	0.11
**R‐4C LVLd**	**cm**	**46, 37, 31**	**8.7 ± 0.6**		**8.5–8.8**	**7**	**8.9 ± 0.7**		**8.7–9.1**	**8**	**8.8 ± 0.7**		**8.6–9.0**	**8**	**0.003**	**0.001**	**0.02**
R‐4C LVLs	cm	46, 36, 31		6.6 [6.2–7.1]	6.4–6.8	12		6.5 [6.0–6.9]	6.3–6.9	10		6.4 [6.2–6.9]	6.3–6.8	9	0.0003	0.001	0.79
**R‐4C SV**	**ml**	**46, 37, 31**	**95.0 ± 23.6**		**89.6–101.3**	**25**	**116.9 ± 30.6**		**109.3–124.8**	**26**	**122.5 ± 33.9**		**112.6–132.3**	**28**	**0.250**	**0.043**	**<0.01**
**R‐4C EF**	**%**	**46, 37, 31**	**58.9 ± 9.5**		**56.6–61.3**	**16**	**63.0 ± 10.0**		**60.2–65.7**	**16**	**61.0 ± 7.7**		**58.8–63.0**	**13**	**0.029**	**0.013**	**0.04**
**R‐LVAdSAX**	**cm** ^**2**^	**46, 37, 31**		**18.8 [17.9–22.1]**	**18.2–20.6**	**18**		**21.4 [19.6–24.3]**	**20.7–22.6**	**17**		**22.2 [19.3–24.7]**	**20.5–22.9**	**16**	**0.025**	**0.005**	**<0.01**
**R‐LVAsSAX**	**cm** ^**2**^	**46, 37, 31**	**6.9 ± 1.9**		**6.4–7.4**	**28**	**7.1 ± 2.1**		**6.6–7.7**	**27**	**7.6 ± 2.6**		**6.9–8.4**	**34**	**0.007**	**0.003**	**0.03**
**R‐LVVdSAX** ^b^	**ml**	**46, 37, 31**		**138.2 [125.0–157.4]**	**137.3–151.9**	**22**		**157.6 [135.3–180.3]**	**154.4–173.6**	**23**		**150.5 [140.1–181.9]**	**155.4–172.7**	**19**	**0.002**	**0.0002**	**<0.01**
R‐LVVsSAX	ml	46, 37, 31	38.6 ± 13.5		35.5–41.8	35	40.2 ± 15.9		36.4–44.8	40	42.0 ± 15.9		37.6–46.9	38	0.038	0.019	0.05
**R‐SV**_**Bullet**_ **SAX** ^b^	**ml**	**46, 37, 31**	**105.8 ± 22.0**		**100.7–111.4**	**21**	**124.1 ± 24.7**		**117.9–130.8**	**20**	**123.1 ± 22.9**		**116.3–129.8**	**19**	**0.002**	**0.0003**	**<0.01**
**Atrium**																	
**R‐4C LAD**	**cm**	**43, 35, 31**	**5.8 ± 0.6**		**5.6–5.9**	**10**	**6.2 ± 0.5**		**6.0–6.3**	**8**	**6.0 ± 0.6**		**5.8–6.1**	**11**	**0.002**	**0.001**	**0.01**
R‐4C LAA	cm2	16, 13, 13	21.4 ± 3.1		20.1–22.6	15	24.1 ± 2.6		23.0–25.2	11	22.9 ± 3.3		21.5–24.4	15	0.014	0.008	0.10
R‐LAD1dSAX	cm	40, 35, 27	4.6 ± 0.5		^a^	11	4.9 ± 0.4		^a^	8	4.5 ± 0.6		^a^	14	0.0004	0.001	0.73
**R‐LAD2dSAX**	**cm**	**38, 34, 27**	**5.1 ± 0.4**		^a^	**8**	**5.3 ± 0.4**		^a^	**7**	**5.4 ± 0.5**		^a^	**9**	**0.003**	**0.001**	**<0.01**
R‐LAAdSAX	cm^2^	15, 13, 12	21.2 ± 3.1		^a^	15	23.2 ± 4.1		^a^	18	23.9 ± 3.2		^a^	13	0.023	0.013	0.09
**Great vessels**																	
R‐AoD_valve_LVOT	cm	24, 22, 14	2.3 ± 0.3		2.2–2.5	14	2.5 ± 0.3		2.4–2.6	12	2.4 ± 0.3		2.3–2.5	12	0.001	0.001	0.23
**R‐AoD**_**sinus**_ **LVOT**	**cm**	**45, 34, 28**	**3.0 ± 0.3**		**3.0–3.1**	**10**	**3.2 ± 0.2**		**3.1–3.2**	**7**	**3.2 ± 0.3**		**3.1–3.3**	**8**	**0.002**	**0.0004**	**<0.01**
**R‐AoD**_**STJ**_ **LVOT**	**cm**	**24, 21, 14**		**2.1 [2.0–2.3]**	**2.0–2.2**	**11**		**2.3 [2.1–2.5]**	**2.2–2.4**	**10**		**2.3 [2.1–2.7]**	**2.2–2.6**	**13**	**0.003**	**0.001**	**<0.01**
R‐PADdLVOT	cm	24, 22, 14	2.0 ± 0.4		1.9–2.1	20	2.0 ± 0.4		1.9–2.1	19	1.8 ± 0.3		1.7–1.9	18	−0.001	0.001	0.05
**R‐PAD**_**valve**_ **RVOT**	**cm**	**29, 31, 22**	**2.6 ± 0.3**		^a^	**12**	**2.5 ± 0.3**		^a^	**14**	**2.5 ± 0.4**		^a^	**16**	**−0.001**	**0.001**	**0.03**
**R‐PAD**_**sinus**_ **RVOT**	**cm**	**24, 24, 16**	**2.8 ± 0.3**		**2.7–2.9**	**11**	**2.7 ± 0.3**		**2.6–2.8**	**10**	**2.5 ± 0.3**		**2.4–2.6**	**10**	**−0.003**	**0.001**	**<0.01**
**R‐AoDdSAX**	**cm**	**42, 35, 28**		**3.3 [3.2–3.5]**	**3.3–3.4**	**8**		**3.4 [3.2–3.6]**	**3.3–3.4**	**8**		**3.6 [3.3–3.7]**	**3.4–3.7**	**9**	**0.002**	**0.0003**	**<0.01**
R‐PAD_valve_SAX	cm	18, 20, 14	2.4 ± 0.3		^a^, ^c^	12	2.5 ± 0.2		^a^, ^c^	8	2.4 ± 0.3		^a^, ^c^	12	−0.001	0.001	0.11
**R‐PAD**_**sinus**_ **SAX**	**cm**	**41, 32, 28**	**2.8 ± 0.3**		^a^, ^c^	**12**	**2.8 ± 0.3**		^a^, ^c^	**9**	**2.7 ± 0.3**		^a^, ^c^	**11**	**−0.002**	**0.001**	**0.03**
**SC AoD**_**sinus**_	**cm**	**15, 19, 15**	**2.9 ± 0.3**		**2.8–3.1**	**10**	**3.1 ± 0.3**		**3.0–3.2**	**10**	**3.1 ± 0.3**		**2.9–3.2**	**10**	**0.002**	**0.001**	**0.03**
**SC Ao**_**CSA**_	**cm** ^**2**^	**15, 19, 15**	**6.9 ± 1.5**		**6.3–7.5**	**22**	**7.5 ± 1.6**		**6.9–8.1**	**21**	**7.5 ± 1.5**		**6.9–8.2**	**20**	**0.009**	**0.004**	**0.02**
**M‐mode**																	
RVIDd	cm	44, 36, 30	2.2 ± 0.6		^c^	27	2.4 ± 0.6		^c^	26	2.4 ± 0.7		^c^	29	0.001	0.001	0.44
IVSd	cm	46, 37,31	1.5 ± 0.2		1.5–1.6	14	1.6 ± 0.2		1.5–1.6	12	1.6 ± 0.2		1.5–1.6	13	0.0003	0.0003	0.35
IVSs	cm	46, 37,31	2.3 ± 0.2		2.2–2.3	10	2.4 ± 0.2		2.3–2.4	9	2.3 ± 0.3		2.2–2.4	11	0.001	0.0004	0.06
**LVIDd** ^b^	**cm**	**46, 37,31**	**5.3 ± 0.6**		**5.2–5.5**	**12**	**5.4 ± 0.6**		**5.3–5.6**	**11**	**5.6 ± 0.8**		**5.4–5.8**	**14**	**0.001**	**0.0002**	**<0.01**
LVIDs	cm	46, 37,31	3.4 ± 0.4		3.3–3.5	12	3.3 ± 0.4		3.2–3.4	13	3.5 ± 0.7		3.3–3.7	20	0.002	0.001	0.06
**LVFWd**	**cm**	**46, 36,31**		**0.9 [0.8–1.0]**	**0.8–0.9**	**18**		**1.0 [0.9–1.1]**	**1.0–1.0**	**17**		**1.0 [0.9–1.2]**	**0.9–1.0**	**19**	**0.002**	**0.0003**	**<0.01**
**LVFWs**	**cm**	**46, 37,31**	**1.6 ± 0.3**		**1.5–1.6**	**17**	**1.8 ± 0.3**		**1.7–1.9**	**17**	**1.8 ± 0.3**		**1.8–1.9**	**16**	**0.003**	**0.001**	**<0.01**
EPSS	cm	43, 37, 30		0.6 [0.5–0.8]	^a^	33		0.6 [0.4–0.7]	^a^	35		0.6 [0.5–0.7]	^a^	33	−0.0002	0.0004	0.55
FS_M‐mode_SAX	%	46, 37,31		37.2 [31.8–40.0]	35.6–38.6	22		38.7 [35.0–43.8]	36.2–41.2	15		37.2 [32.6–42.9]	33.7–38.6	20	0.020	0.015	0.19
EF_M‐mode_SAX	%	46, 37,31		66.6 [60.0–69.7]	65.0–68.6	19		68.7 [63.5–74.6]	65.1–71.8	10		66.0 [59.6–73.2]	62.5–68.5	13	0.023	0.021	0.27
**EDV**_**M‐mode**_ **SAX** ^b^	**ml**	**46, 37,31**		**131.1 [111.3–150.5]**	**129.6–148.1**	**29**		**132.0 [115.8–158.3]**	**134.1–155.4**	**27**		**155.6 [120.3–194.5]**	**144.6–176.4**	**33**	**0.002**	**0.0004**	**<0.01**
**ESV**_**M‐mode**_ **SAX**	**ml**	**46, 37,31**	**47.2 ± 14.4**		**43.6–50.8**	**30**	**44.3 ± 13.5**		**40.9–48.3**	**31**	**54.8 ± 24.4**		**48.0–62.7**	**44**	**0.066**	**0.028**	**0.02**
**MASS**_**M‐mode**_ **SAX**	**g**	**24, 24, 16**	**263.9 ± 54.9**		**246.7–280.9**	**21**	**303.3 ± 66.5**		**281.9–325.5**	**22**	**323.9 ± 63.3**		**298.3–349.5**	**20**	**0.584**	**0.109**	**<0.01**
RWT_M‐mode_SAX		46, 37,31		0.6 [0.5–1.0]	0.5–0.9	43		0.5 [0.5–1.0]	0.5–0.6	45		0.7 [0.5–1.1]	0.5–1.0	44	0.0003	0.0002	0.17
**SV**_**M‐mode**_ **SAX** ^b^	**ml**	**46, 37,31**		**83.6 [69.6–106.9]**	**84.5–99.0**	**34**		**94.5 [81.6–109.9]**	**92.4–109.2**	**31**		**96.9 [84.5–128.3]**	**95.3–114.4**	**32**	**0.002**	**0.001**	**<0.01**
**Doppler**																	
**R‐PA Vel**_**max**_ **RVOT**	**m/s**	**28, 30, 19**	**1.0 ± 0.1**		**1.0–1.0**	**14**	**1.2 ± 0.2**		**1.2–1.2**	**16**	**1.2 ± 0.2**		**1.2–1.3**	**14**	**0.209**	**0.038**	**<0.01**
**R‐PA PG RVOT**	**mmHg**	**28, 30, 19**	**4.1 ± 1.1**		**3.7–4.4**	**28**	**5.5 ± 0.2**		**5.0–6.0**	**32**	**6.0 ± 1.7**		**5.4–6.6**	**28**	**0.018**	**0.004**	**<0.01**
**R‐PA Vel**_**max**_ **SAX**	**m/s**	**29, 21, 18**	**1.0 ± 0.2**		^c^	**17**	**1.2 ± 0.2**		^c^	**15**	**1.2 ± 0.2**		^c^	**15**	**0.229**	**0.044**	**<0.01**
**R‐PA PG SAX**	**mmHg**	**28, 21, 17**	**3.9 ± 1.3**		**3.5–4.3**	**33**	**5.5 ± 1.8**		**4.9–6.1**	**32**	**5.8 ± 1.6**		**5.2–6.5**	**28**	**0.020**	**0.004**	**<0.01**
R‐4C TV_E_	m/s	27, 24, 19	0.6 ± 0.1		0.6–0.7	19	0.7 ± 0.2		0.6–0.7	23	0.6 ± 0.1		0.6–0.7	16	0.028	0.033	0.40
R‐4C TV_A_	m/s	27, 24, 19		0.5 [0.5–0.6]	^a^, ^c^	21		0.5 [0.5–0.6]	^a^, ^c^	16		0.5 [0.5–0.6]	^a^, ^c^	16	−0.022	0.026	0.41
R‐4C TV_E_/TV_A_		27, 24, 19	1.2 ± 0.3		1.1–1.2	22	1.2 ± 0.3		1.2–1.3	22	1.2 ± 0.22		1.1–1.3	19	0.001	0.001	0.21
**SC Ao Vel**_**max**_	**m/s**	**24, 24, 15**	**1.6 ± 0.3**		**1.6–1.7**	**17**	**1.9 ± 0.3**		**1.8–2.0**	**16**	**1.9 ± 0.3**		**1.7–2.0**	**18**	**0.220**	**0.080**	**0.01**
**SC Ao VTI**	**cm**	**24, 24, 15**	**29.1 ± 4.7**		**27.6–30.7**	**16**	**32.6 ± 4.5**		**31.1–34.0**	**14**	**32.5 ± 4.8**		**30.5–34.4**	**15**	**0.033**	**0.011**	**0.01**
**SC Ao PG**	**mmHg**	**24, 24, 15**	**11.1 ± 3.9**		**9.9–12.5**	**35**	**15.0 ± 4.4**		**13.5–16.3**	**29**	**14.2 ± 4.7**		**12.1–16.0**	**33**	**0.031**	**0.012**	**0.01**
**SC Ao SV**	**ml**	**15, 19, 15**	**209.0 ± 63.2**		**184.7–235.3**	**30**	**247.9 ± 64.9**		**224.5–271.2**	**26**	**242.4 ± 52.7**		**221.1–264.8**	**22**	**0.316**	**0.149**	**0.04**
**SC MV**_**E**_	**m/s**	**22, 24, 14**	**0.8 ± 0.1**		**0.7–0.8**	**16**	**1.0 ± 0.1**		**0.9–1.0**	**14**	**0.9 ± 0.1**		**0.9–1.0**	**14**	**0.151**	**0.043**	**<0.01**
SC MV_A_	m/s	22, 24, 14		0.6 [0.5–0.7]	0.6–0.7	19		0.6 [0.5–0.9]	0.6–0.8	26		0.6 [0.5–0.7]	0.5–0.6	25	−0.0004	0.001	0.57
**SC MV**_**E**_ **/MV**_**A**_		**22, 24, 14**	**1.2 ± 0.3**		**1.2–1.3**	**23**	**1.5 ± 0.4**		**1.4–1.6**	**27**	**1.6 ± 0.4**		**1.4–1.8**	**26**	**0.002**	**0.001**	**0.01**

*Note*: Parametric data are represented as mean ± standard deviation (SD), and non‐parametric data as median [interquartile range (IQR)]. Parameters that presented significant changes over time are shown in bold. Abbreviations and imaging and measurement details are available in Table S1.

^a^ Parameter with inter‐observer intraclass correlation coefficient <0.60.

^b^ Variable log‐transformed for linear mixed model analysis.

^c^ Parameter with intra‐observer intraclass correlation coefficient <0.60.

## 4 DISCUSSION

Overall, our findings demonstrate a gradual adaptation of the cardiorespiratory system from foetal to extrauterine life in parallel with body growth and age, as expected in healthy neonatal foals. This is reflected by the progressive increase in left ventricular and atrial dimensions, as well as aortic diameter, alongside a decrease in pulmonary artery diameter over time.

Similar progressive increases in left ventricular and atrial dimensions have been observed in human neonates during the first weeks of life, reflecting the transition from foetal to postnatal circulation.^23^, ^24^ This adaptation is driven by increased pulmonary blood flow, closure of foetal cardiac shunts (ductus arteriosus and foramen ovale) and the need for the left heart to accommodate rising systemic venous return, ensuring sufficient stroke volume and cardiac output as the neonate grows.^23^, ^25^

Neonatal foals exhibit a wide physiological range of heart rates, which can vary even within the time groups considered herein.^18^ Although heart rate assessment was not the primary focus of this study, the wide heart rate range observed (from 60 to 138 bpm) may have influenced the higher coefficients of variations observed for some echocardiographic variables, reflecting physiological variability rather than measurement error.^26^

Quantification of cardiac size and function in horses is mostly performed by echocardiography, although it relies on operator technique, equipment capacity, adequate acoustic windows and geometric assumptions regarding heart shape. To our knowledge, only one study employing another imaging modality (magnetic resonance) has been reported.^27^ Magnetic resonance is considered the reference standard for evaluation of cardiac morphology and measurement of cardiac function in other species.^28^ In a study with anaesthetised 5‐ to 9‐day‐old foals, 2DE underestimated left ventricular end‐diastolic and end‐systolic volume and overestimated left ventricular ejection fraction when compared with magnetic resonance.^27^ Even though the employment of such advanced imaging techniques is not feasible in routine equine cardiology, studies like this are important to elucidate the limits of more conventional techniques and aid in their interpretation. Foals from that study were older and substantially heavier than those herein. Also, foals were anaesthetised, which might interfere in volumetric indexes such as stroke volume.^29^ Altogether, those differences preclude a meaningful comparison of measurements.

The only echocardiographic study that performed repeated assessments in a similar age group of foals as the present study was performed by Stewart et al. in ponies and Thoroughbred foals.^13^ Notably, the ultrasound machines and techniques employed at that time were different from those available nowadays, hindering direct comparisons. Nevertheless, that study also found a decrease in the ratio between left atrium and aorta diameter starting at 24 h after birth. By then, it was suggested that it could be due to foramen ovale and ductus arteriosus closure. Nowadays, with further studies assessing more profoundly those structures, it is well known that their closure occurs much later, even in healthy foals.^30^, ^31^ In our study population, flow through both foramen ovale and ductus arteriosus was regularly observed regardless of age.

Lombard et al. assessed 12 pony foals with echocardiography, electrocardiography, blood pressure, heart rate and auscultation at 1, 7, 14, 21, 30, 60 and 90 days of life.^12^ A proportional growth of ventricular dimensions and aortic root diameter during the first weeks of life was observed, corroborating our results. The fractional shortening reported by Lombard et al. and in subsequent studies on older foals was comparable to the values observed in the present study.^7^, ^12^, ^16^

As mentioned before, an increase in left ventricular dimensions and estimated weight over time was observed in the present study. Machida et al. reported a gradual increase in the ratio of the right ventricular weight to the total ventricular weight in equine foetus from 190 to 330 days of pregnancy.^32^ This was followed by a decrease in this ratio in newborn foals immediately after birth and in the subsequent 11 days. Also, a significant increase (49.5%) in left ventricle and septum weight was observed in the same period, along with a much slower rate of weight gain in the right ventricle (19.1%). Together, these observations highlight the asymmetric growth of ventricular components during the perinatal period in foals, likely reflecting the cardiovascular adjustments required for the transition from foetal to neonatal circulation. Finally, the increase in left ventricular dimensions likely accounts for the observed rise in stroke volume, allowing for greater end‐diastolic filling.

Very little information is available regarding the optimal echocardiographic method to measure stroke volume and cardiac output in foals. Giguère et al., in a study with anaesthetised neonatal foals, compared several volumetric and Doppler techniques to lithium dilution and identified the Bullet method as the most accurate for cardiac output measurement.^33^ In line with this, our preliminary observations suggest that volumetric methods (Simpson, Bullet and Teichholz) differ considerably from Doppler‐based assessment through the aortic flow in the subcostal view (unpublished data).

In our study we performed one measurement of the right ventricle (RVIDd) but due to its fair intra‐observer reliability (ICC = 0.57), this parameter was not considered for further analysis. Accurate measurements of right ventricular dimensions in horses are challenging due to its complex anatomy, thin trabeculated walls and susceptibility to loading conditions, respiratory variation, transducer placement and imaging plane.^34^ Other measurements, such as right ventricle area in various views, fractional area change and tissue Doppler indices, have been described in adult horses,^34^ but their feasibility and reliability in foals remain unknown. The subcostal view could also aid in the assessment of the right heart in foals, but it has not been systematically evaluated for that purpose.

An increase over time was observed in left atrium diameter in the right long and short axis views (R‐4C LAD and R‐LAD2dSAX). This suggests a normal physiological transition from foetal to neonatal circulation, where the increase in left atrial dimensions is likely driven by enhanced pulmonary circulation and the resulting volume load as the heart adapts to the increased circulatory demands of postnatal life.^2^ This is further supported by the increase in pulmonary flow peak velocity over time, observed both in SAX and RVOT.

In the present study, the pressure gradient in the pulmonary artery increased over time. This likely reflects the previously mentioned rise in pulmonary blood flow velocity, as the pressure gradient is flow‐dependent and proportional to the square of velocity.^35^ These haemodynamic changes are consistent with the initiation of respiration at birth, which induces pulmonary vasodilation, reduces pulmonary vascular resistance and facilitates a marked increase in pulmonary blood flow.^23^ A postnatal decrease in pulmonary artery systolic pressure was observed in healthy human newborns.^36^, ^37^

Many of the parameters with fair and poor ICCs in the intra and interobserver assessments are derived from the short axis view of the heart base, which very often yields images with poorer quality and has intrinsic anatomic limitations in horses.^38^ RVIDd and EPSS, which in this study presented fair agreement in intra‐ and inter‐observer assessments, respectively, have also presented high variability in a previous study in foals,^14^ confirming they should be interpreted with caution.

Often statistical differences were observed, but numerical differences were as small as 0.1 cm. Interpretation of this is challenging, especially in an echocardiography context, so care must be taken not to overestimate their meaning. Similarly, some limitations of the present study must be highlighted. First, and inherent to echocardiographic studies in the equine species, there is an inaccuracy of measurements compared with the actual structures assessed and the inability to measure all cardiac chambers. In addition, measurements for some variables are lacking due to poor image quality. This could have been caused by movements during the exam, a small thoracic size that hindered optimal probe positioning, high heart rate and occasional lack of adequate contact between the probe and the thoracic wall. Second, CoVs are moderate to high in a considerable number of variables. This can be attributed to multiple factors like the employment of two different ultrasound machines to perform the study (as CoVs were not calculated separately per machine) and heart rate variations between foals, even within the same echocardiographic exam. Also, the operator gained more experience over time. Furthermore, the timing of echocardiographic assessments spanned up to 48 h per period, creating irregular sampling which may have influenced analyses, as this is a time‐dependent study. Moreover, it was not possible to determine to what extent the increase in cardiac structures was attributable to physiological cardiovascular adaptation versus normal body growth. In this study, echocardiographic measurements were not indexed to bodyweight as included foals had very similar bodyweights, with minimal variability among individuals. An additional consideration should be made on frame rate, which was not included among the examined variables as the primary focus was on measurement reproducibility and biological variation rather than technical parameters. However, it is important to consider that image acquisition settings were kept as consistent as possible throughout echocardiographic examinations. The Clinical and Standards Laboratory Institute guidelines recommend having a reference population of at least 120 subjects.^39^ Regrettably, reaching such a high number of healthy subjects is particularly challenging in equine medicine, especially in the context of foals. However, it should be noted that the present study represents the one with the largest sample size in equine early neonatal echocardiography and does not aim to establish reference intervals. Lastly, mitral valve flow assessment was performed through a non‐validated technique (subcostal view). In the authors' experience, this modality allows for a non‐inferior alignment between the ultrasound beam and the transmitral flow (Figure S1). An appraisal of its diagnostic reliability has been submitted by the authors and is currently under review.

In summary, these findings support the interpretation of a gradual and physiological adaptation of the cardiorespiratory system from foetal to extrauterine life, reflected by a progressive increase in left ventricular, left atrial and aortic diameters, alongside a decrease in pulmonary artery diameter over time. Some parameters, such as R‐4C LVVs, RVIDd, EPSS, R‐4C LAD, R‐LAD1dSAX, R‐LAD2dSAX, R‐LAAdSAX, R‐PAD_valve_RVOT, R‐PAD_valve_SAX, R‐PAD_sinus_SAX, R‐PA Vel_max_ SAX and R‐4C TV_A_, should be interpreted with caution as intra‐ and/or inter‐observer variability is higher. We believe these results offer a relevant contribution to foal cardiology by improving the interpretation of neonatal cardiac findings and advancing the understanding of early cardiocirculatory adaptation in horses. Additionally, they may serve as a valuable baseline for future echocardiographic studies in sick foal populations.

## AUTHOR CONTRIBUTIONS

**Fernanda Timbó D'el Rey Dantas:** Investigation; writing – original draft; methodology; writing – review and editing; visualization; data curation. **Giulia Forni:** Investigation; writing – review and editing; methodology; data curation; writing – original draft. **Gayle Hallowell:** Writing – review and editing; methodology; validation; data curation. **Carolina Castagnetti:** Writing – review and editing; resources; validation. **Laura Menchetti:** Formal analysis; data curation; validation; writing – review and editing. **Giovanni Romito:** Writing – review and editing; methodology; validation; data curation. **Aliai Lanci:** Writing – review and editing; resources; investigation. **Jole Mariella:** Writing – review and editing; resources; investigation. **Francesca Freccero:** Methodology; conceptualization; investigation; writing – review and editing; project administration; supervision; validation; data curation.

## FUNDING INFORMATION

No funding was received.

## CONFLICT OF INTEREST STATEMENT

The authors have declared no conflicting interests.

## DATA INTEGRITY STATEMENT

Francesca Freccero had full access to all the data in the study and takes responsibility for the integrity of the data and the accuracy of data analysis.

## ETHICAL ANIMAL RESEARCH

Approved by the Animal Welfare Committee of Bologna University (4646/2024).

## INFORMED CONSENT

Informed consent for use of the study subjects was provided by owners.

## Supporting information


**Figure S1.** Mitral valve flow assessment from the subcostal view in a healthy Standardbred neonatal foal. LA, left atrium; LV, left ventricle; MV, mitral valve.

**Table S1.** Echocardiographic views, methods, abbreviations and units of measurements and indices used in the present study.

## Data Availability

The data that support the findings of this study are openly available in the Institutional Research Repository of the University of Bologna at http://doi.org/10.6092/unibo/amsacta/8677.
