# Supplementary material for: Mono‐dimensional, two‐dimensional and Doppler echocardiographic measurements in healthy Standardbred neonatal foals in the first 5 days of life
Source: Equine Vet J. 2026 Jan 6;58(5):1345–54. doi: 10.1002/evj.70140 (PMC13447962; doi:10.1002/evj.70140)

Equine Veterinary Journal BEVA

Figure S1: Mitral valve flow assessment from the subcostal view in a healthy Standardbred neonatal foal. LV: left ventricle; MV: mitral valve; LA: left atrium.

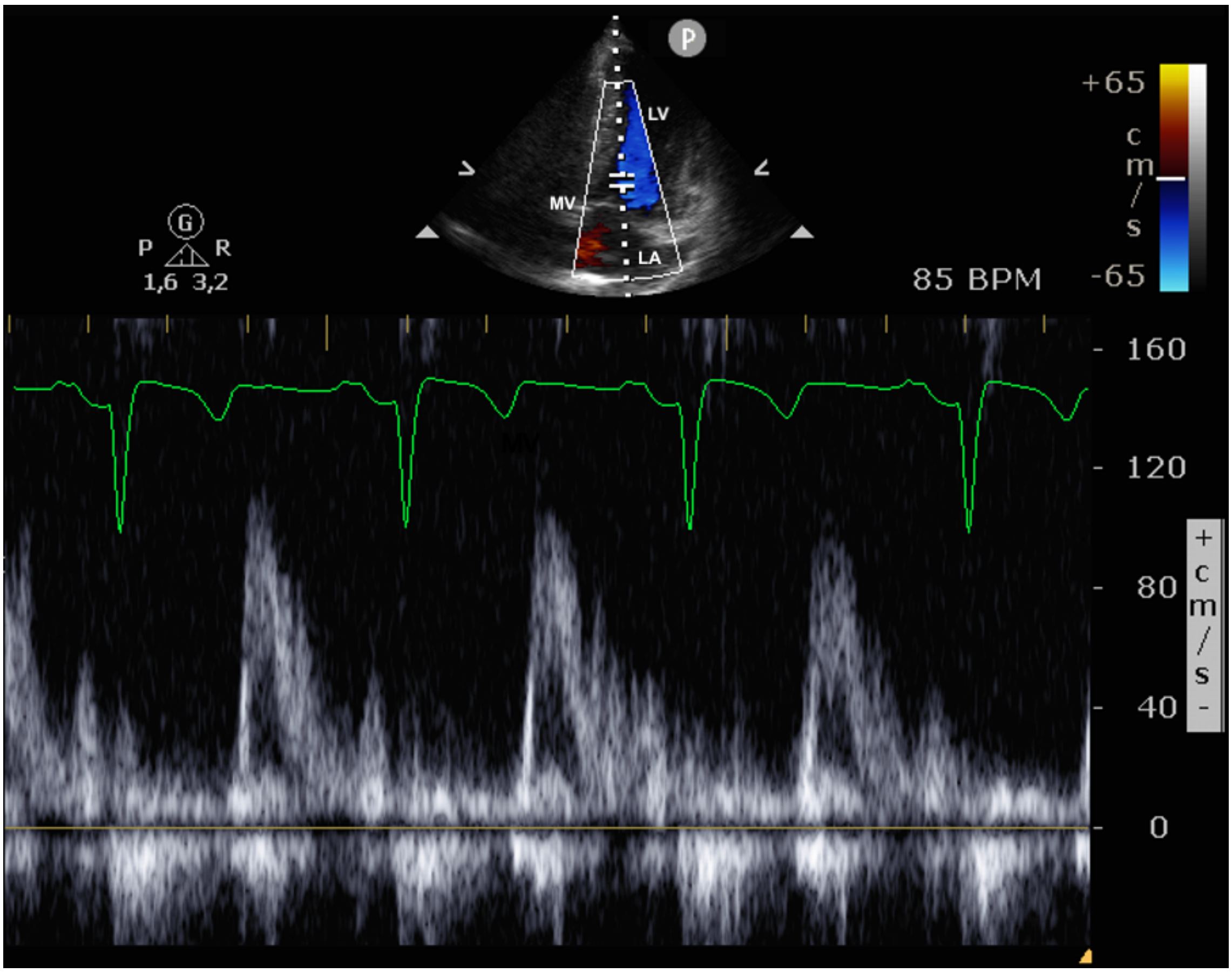

Supplement: Supplementary file 1 [file EVJ-58-1345-s001.pdf]
