# Supplementary material for: Mono‐dimensional, two‐dimensional and Doppler echocardiographic measurements in healthy Standardbred neonatal foals in the first 5 days of life
Source: Equine Vet J. 2026 Jan 6;58(5):1345–54. doi: 10.1002/evj.70140 (PMC13447962; doi:10.1002/evj.70140)
Supplement: Supplementary file 2 [file EVJ-58-1345-s002.pdf]

Equine Veterinary Journal

BEVA

Table S1: Echocardiographic views, methods, abbreviations and units of measurements and indices used in the present study.

Scan view	Abbreviation	Unit	Measurement method
Right-sided parasternal long-axis 4-chamber view (R-4C)	R-4C LVVd	ml	Left ventricular volume measured at end-diastole, timed one frame after mitral valve closure
	R-4C LVVs	ml	Left ventricular volume measured at end-systole, timed one frame prior to mitral valve opening
	R-4C LVLd	cm	Left ventricular length measured at end-diastole, timed one frame after mitral valve closure
	R-4C LVLs	cm	Left ventricular length measured at end-systole, timed one frame prior to mitral valve opening
	R-4C LAD	cm	Left atrium diameter at the widest distance parallel to the mitral valve annulus during maximum atrial filling
	R-4C LAA	cm^2	Left atrial area at maximum atrial filling
	R-4C TV_E	m/s	Transtricuspid flow measured at the peak of E wave
	R-4C TV_A	m/s	Transtricuspid flow measured at the peak of A wave
	R-4C SV	ml	Stroke volume calculated by the modified Simpson's method.
	R-4C EF	%	Left ventricular ejection fraction calculated by the modified Simpson's method.
Right-sided parasternal long-axis right ventricular outflow tract view (R-RVOT)	R-PAD_{valve}RVOT	cm	Pulmonary diameter at the valve level at end-diastole
	R-PAD_{sinus}RVOT	cm	Pulmonary diameter at the sinus at end-diastole
	R-PA Vel_{max}RVOT	m/s	Pulmonary artery peak velocity
	R-PA PG RVOT	mmHg	Pulmonary artery pressure gradient
Right-sided parasternal long-axis left ventricular outflow tract view (R-LVOT)	R-AoD_{valve}LVOT	cm	Aorta diameter at valve level at end-diastole, one frame before valve opening
	R-AoD_{sinus}LVOT	cm	Aorta diameter at sinus at end-diastole, one frame before valve opening
	R-AoD_{STJ}LVOT	cm	Aorta diameter at sinotubular junction at end-diastole, one frame before valve opening
	R-PADdLVOT	cm	Pulmonary artery diameter measured at end-diastole
	R-LVAdSAX	cm^2	Left ventricular area measured at end-diastole
	R-LVAsSAX	cm^2	Left ventricular area measured at peak-systole
	R-LVVdSAX	ml	Left ventricular volume measured at end-diastole, timed one frame after mitral valve closure

Right-sided short axis view (R-SAX)	R-LVVsSAX	ml	Left ventricular volume measured at end-systole, timed one frame prior to mitral valve opening
	R-SV_{Bullet}SAX	ml	Stroke volume calculated by the bullet method.
	RVIDd	cm	Right ventricle internal diameter measured in M-mode at end-diastole
	IVSd	cm	Interventricular septal thickness measured in M-mode at end-diastole
	IVSs	cm	Interventricular septal thickness measured in M-mode at peak-systole
	LVIDd	cm	Left ventricular internal diameter in M-mode at end-diastole
	LVIDs	cm	Left ventricular internal diameter in M-mode at peak-systole
	LVFWd	cm	Left ventricular free wall thickness in M-mode at end-diastole
	LVFWs	cm	Left ventricular free wall thickness in M-mode at peak-systole
	EPSS	cm	E-point septal separation in M-mode at the level of the mitral valve
	$FS_{M\text{-}mode}$SAX	%	Left ventricular fractional shortening calculated using M-mode measurements
	$EF_{M\text{-}mode}$SAX	%	Left ventricular ejection fraction calculated using M-mode measurements
	$EDV_{M\text{-}mode}$SAX	ml	End diastolic volume calculated using M-mode measurements
	$ESV_{M\text{-}mode}$SAX	ml	End systolic volume calculated using M-mode measurements
	$MASS_{M\text{-}mode}$SAX	g	Left ventricular mass calculated using M-mode measurements
	$RWT_{M\text{-}mode}$SAX		Left ventricular relative wall thickness calculated using M-mode measurements
	$SV_{M\text{-}mode}$SAX	ml	Stroke volume calculated using M-mode measurements
	R-AoDdSAX	cm	Aorta diameter at level of the base of the heart at diastole
	R-LAD1dSAX	cm	Left atrium diameter from the commissure of the left coronary and noncoronary cusps to the opposite atrial wall at diastole
	R-LAD2dSAX	cm	Left atrium diameter from the junction of the atrial septum with aorta to the free wall at diastole
	R-LAAdSAX	cm^2	Left atrium area at diastole
	R-PAD_{valve}SAX	cm	Pulmonary artery diameter at the valve level at diastole
	R-PAD_{sinus}SAX	cm	Pulmonary artery diameter at the sinus of Valsava at diastole
	R-PA Vel_{max} SAX	m/s	Pulmonary flow maximal velocity
	R-PA PG SAX	mmHg	Pulmonary flow pressure gradient
Subcostal view (SC)	SC AoD_{sinus}	cm	Aorta diameter at the sinus of Valsava at end-diastole
	SC Ao_{CSA}	cm^2	Aorta cross sectional area calculated from SC AoD_{sinus}
	SC Ao VTI	cm	Aorta flow velocity time integral
	SC Ao Vel_{max}	m/s	Aorta flow maximal velocity

	SC Ao PG	mmHg	Aorta flow pressure gradient
	SC Ao SV	ml	Stroke volume calculated using aorta flow velocity time integral and cross-sectional area
	SC MV_E	m/s	Transmitral flow measured at the peak of E wave
	SC MV_A	m/s	Transmitral flow measured at the peak of A wave

References: [8, 33, 35, 38, 40-43]
